# Effects of Prepartum Supplementation with Flaxseed and Black Soldier Fly Larvae Oils on Fatty Acid Composition and Lipid Quality Indices in Sheep Milk

**DOI:** 10.3390/molecules31152628

**Published:** 2026-07-28

**Authors:** Kamila Lewandowska, Natalia Pachura-Hanusek, Antoni Szumny, Anna Zielak-Steciwko, Robert Kupczyński

**Affiliations:** 1 Department of Environment Hygiene and Animal Welfare, Faculty of Biology and Animal Science, Wrocław University of Environmental and Life Sciences, Chełmońskiego St. 38c, 51-630 Wrocław, Poland; natalia.pachura@upwr.edu.pl; 2 Department of Food Chemistry and Biocatalysis, Faculty of Biotechnology and Food Science, Wrocław University of Environmental and Life Sciences, Norwida St. 25, 50-375 Wrocław, Poland; antoni.szumny@upwr.edu.pl; 3 Institute of Animal Husbandry and Breeding, Faculty of Biology and Animal Science, Wrocław University of Environmental and Life Sciences, Chełmońskiego St. 38c, 50-375 Wrocław, Poland; anna.zielak-steciwko@upwr.edu.pl

**Keywords:** sheep milk, black soldier fly larvae oil, flaxseed oil, fatty acids, conjugated linoleic acid, mature milk, transitional milk

## Abstract

The peripartum period in prolific ewes is associated with increased metabolic demands, and dietary lipid supplementation may increase the energy density of the prepartum diet. This study evaluated the effects of prepartum supplementation with alternative lipid matrices on the physicochemical characteristics and comprehensive fatty acid (FA) profiles of mature and transitional sheep milk. Thirty-two pregnant Olkuska ewes were randomly assigned to four cohorts (*n* = 8/group): Control (basal diet), flaxseed oil (low n-3/high n-6 variety; FO), black soldier fly larvae oil (BSFL oil), and Mix oil (2:1 ratio of FO to BSFL oil). Dietary treatments exerted no significant effects (*p* > 0.05) on transitional milk composition or lipid quality indices. In contrast, in mature milk, BSFL oil supplementation significantly increased milk fat content (6.09%) compared to the Control group (4.70%; *p* < 0.01). Furthermore, all oil treatments significantly reduced total saturated FAs (*p* < 0.01), while the BSFL oil and Mix oil supplementation significantly increased the monounsaturated FA fraction (*p* < 0.01). The differences, particularly the increase in *cis*-9, *trans*-11 CLA concentration and the significant reduction in both the atherogenic and thrombogenic indices (*p* < 0.01), were observed exclusively in mature milk. These findings suggest that the combination of sustainable insect lipids with high-linoleic plant oils may represent a viable strategy to transform sheep milk into a functional food with potential cardiovascular health benefits for consumers. These findings additionally indicate that prepartum lipid supplementation may exert a persistent carry-over effect into early lactation, influencing the fatty acid composition of mature sheep milk.

## 1. Introduction

In the landscape of functional foods, dairy products derived from small ruminants, particularly sheep, occupy a distinct position. Beyond its highly bioavailable protein and high mineral content, sheep milk is a complex lipid matrix naturally rich in branched-chain fatty acids and conjugated linoleic acid (*cis*-9, *trans*-11 CLA) [1,2]. These specific lipid fractions have drawn considerable interest in human nutrition due to their potential anti-inflammatory, anti-atherogenic, and metabolic effects [2,3,4,5]. However, the fatty acid profile of ewe milk is not constant and is influenced by maternal diet, ruminal fermentation kinetics, and the specific stage of lactation [6,7,8,9].

The peripartum period, extending from late gestation to early lactation, is characterized by substantial metabolic and physiological changes in the production cycle of dairy ewes [10,11]. The onset of colostrogenesis and subsequent milk synthesis markedly increase energy and nutrient requirements, which may contribute to a negative energy balance [12,13,14,15]. Lipid supplementation during late gestation can increase the density of dietary energy and modify the supply of fatty acids available for ruminal metabolism, tissue deposition, and subsequent secretion in milk [16,17].

Traditionally, dietary strategies aimed at modifying milk fat have relied on conventional plant oils to shift the polyunsaturated fatty acid (PUFA) profile [18,19,20,21]. The low-linolenic/high-linoleic flaxseed oil used in the present study differs from conventional flaxseed oil because linoleic acid (LA, C18:2 n-6, *cis*), rather than α-linolenic acid (ALA, C18:3 n-3), is its predominant polyunsaturated fatty acid. In the rumen, LA undergoes biohydrogenation, generating intermediates such as *trans*-11 C18:1 (vaccenic acid) and *cis*-9, *trans*-11 CLA. Vaccenic acid may subsequently be taken up by the mammary gland and converted to *cis*-9, *trans*-11 CLA [22,23]. Currently, the growing interest in sustainable livestock production has increased attention to circular feed resources. Insect larvae oils (such as those of *Hermetia illucens* or *Tenebrio molitor*) may represent alternative sources of feed lipids that reduce reliance on crops also used for human food [24,25,26]. In contrast to high-linoleic plant oils, insect-derived lipids provide a markedly different fatty acid profile and, therefore, may exert distinct effects on ruminal lipid metabolism and milk fat composition. Chemically, the oil of Black Soldier Fly larvae is typically rich in medium-chain fatty acids (MCFAs) such as lauric acid (C12:0), alongside varying levels of oleic acid [27,28,29]. Blending a high-linoleic modified plant oil with an insect-derived lipid source creates a mixture of lipid sources with contrasting fatty acid profiles that may be metabolized differently in the rumen; however, how this combination affects the maternal transition into lactation remains largely unexplored. To date, the use of Black Soldier Fly larvae oil in prepartum dairy ewe diets, particularly in combination with high-linoleic flaxseed oil, has not been adequately investigated. Accordingly, the novelty of the present study lies in the prepartum timing of supplementation, the use of Black Soldier Fly larvae oil alone and in a binary blend with high-linoleic flaxseed oil, and the evaluation of whether treatment-associated differences remained detectable in mature milk after supplementation had ceased.

Although the dietary manipulation of mature sheep milk fat during established lactation is well documented, data on the critical transitional phase from colostrum to mature milk remain surprisingly sparse [30,31,32]. This period represents a profound physiological transition during which the gross chemical composition and fatty acid profile of milk change rapidly on a daily basis. Understanding whether prepartum supplementation with high-linoleic flaxseed oil and black soldier fly larvae oil can influence these early changes is important for assessing its effects on milk composition and the concentration of nutritionally relevant fatty acids. To address this knowledge gap, the present study evaluated the effects of prepartum supplementation with high-linoleic flaxseed oil, Black Soldier Fly larvae oil, and their binary blend on the basic composition and comprehensive fatty acid profile of transitional and mature sheep milk. The primary objective was to determine whether prepartum supplementation with these contrasting lipid sources modifies the composition and fatty acid profile of transitional and mature milk and whether these treatment-associated effects remain detectable after supplementation ceased at lambing.

## 2. Results

### 2.1. Basal Diet and Supplement Composition

The chemical composition and mineral content of the basal diet components provided to the sheep during the experiment are shown in Table 1. The basal diet consisted of meadow hay and a custom-made concentrate mixture containing 50% oats, 30% triticale, 10% soybean meal, 5% maize, and 5% mineral-vitamin premix (Polfamix). Both feed components exhibited standard dry matter content (DM), 92.83% for hay and 90.62% for the concentrate mixture.

The analytical values obtained for both components were consistent with the standard nutritional requirements for sheep. The concentrate mixture served as the primary source of protein and energy, containing 16.96% DM of crude protein and 9.61% DM of crude fat, alongside a low fiber content (11.51% DM of crude fiber and 33.79% DM of NDF). In contrast, the meadow hay provided the necessary structural fiber, with crude fiber, NDF, and ADF fractions determined at 32.34%, 59.85% and 23.23% DM, respectively.

Regarding the macromineral profile, the concentrate mixture had a calcium (Ca) content of 21.89 g/kg DM and a phosphorus (P) content of 5.71 g/kg DM (Ca:P ratio of 3.84:1), reflecting the addition of the mineral-vitamin premix. The hay contained 6.47 g/kg DM of Ca and 2.40 g/kg DM of P, maintaining a physiologically appropriate Ca:P ratio of 2.70:1.

#### 2.1.1. Fatty Acid Profile (% of Total FAs) of the Basal Diet Components

The comprehensive fatty acid composition of the basal diet components (meadow hay and concentrate mixture) is detailed in Table 2. The evaluated feedstuffs showed different fatty acid compositions, reflecting their distinct plant origins and structural processing.

The meadow hay lipid matrix was predominantly saturated (56.66% SFA), driven primarily by palmitic acid (C16:0; 38.29%), followed by stearic (C18:0; 7.48%) and lignoceric (C24:0; 5.20%) acids. Polyunsaturated fatty acids (PUFA) accounted for 36.95% of the total forage lipids, maintaining a balanced distribution of linoleic (C18:2 n-6, *cis*; 19.44%) and α-linolenic (C18:3 n-3; 17.51%) acids, which resulted in a tight forage n-6/n-3 ratio of 1.11.

Conversely, the concentrate mixture was characterized by a high content of unsaturated fatty acids (77.23% UFA), with a clear predominance of PUFAs (49.63%). This matrix was characterized by a high concentration of linoleic acid (44.60%), alongside a substantial monounsaturated fraction (27.60% MUFA) driven almost entirely by oleic acid (C18:1 *cis*-9; 27.02%). Due to the components of cereal and oilseed meal, the n-3 PUFA share was low (5.03%), resulting in an elevated dietary ratio of 8.87. The saturated fractions in the concentrate were minor (23.23%), with palmitic acid as the main compound (17.25%).

#### 2.1.2. Fatty Acid Profile of the Experimental Oils

The fatty acid composition of a selected low n-3/high n-6 flaxseed oil and BSFL oil is presented in Table 3. The analyzed flaxseed oil exhibited a lipid profile atypical of conventional flaxseed oils, with a clear predominance of n-6 PUFAs. The principal fatty acid identified in the sample was linoleic acid (C18:2 n-6, *cis*), which accounted for 54.66% of total FAs. Consistent with the characteristics of this variety, a drastically reduced content of α-linolenic acid (C18:3 n-3) was observed, which was only 2.96%. Among the other significant fatty acid fractions, palmitic acid (C16:0; 16.26%), oleic acid (C18:1 *cis*-9; 13.60%), and stearic acid (C18:0; 11.30%) were identified.

In contrast to the flaxseed oil, the lipid profile of the insect fat was strongly dominated by saturated fatty acids (SFA), with a particular emphasis on medium-chain fatty acids (MCFA). The most abundant fraction was lauric acid (C12:0), with a concentration averaging 51.93% of total FAs. Furthermore, high contents of other saturated fatty acids were identified in this matrix: palmitic acid (C16:0; 19.38%) and myristic acid (C14:0; 11.72%). The proportion of unsaturated fatty acids in insect fat was relatively low, with the highest contents recorded for linoleic acid (C18:2 n-6, *cis*; 5.34%) and oleic acid (C18:1 *cis*-9; 5.25%).

### 2.2. Transitional and Mature Milk Composition

Regarding the transitional milk, the dietary supplementation with different oil sources (flaxseed, BSFL and Mix) did not exert any statistically significant effect on its physicochemical parameters (*p* > 0.05, Table 4). The fat content remained stable across all groups, ranging from 6.66% in the Mix oil group to 7.42% in the Control group (*p* = 0.515). Similarly, no significant alterations were observed for SNF (*p* = 0.861), density (*p* = 0.964), total protein (*p* = 0.860), lactose (*p* = 0.858), and mineral salts content (*p* = 0.804). Individual ewe-level values are presented in Figure S1. An observation in the FO group was flagged by the 1.5 × IQR rule, but it was retained because no analytical or data entry error was identified.

Unlike transitional milk, the physicochemical profile of mature milk was significantly influenced by the source of dietary oil. A significant effect was observed for fat content (*p* = 0.008). Supplementation with insect oil (BSFL oil) resulted in a significantly higher milk fat percentage (6.09%) compared with the Control group (4.70%; *p* < 0.01). The FO and Mix oil groups had intermediate fat contents of 5.55% and 5.68%, respectively. These values did not differ statistically significantly from that of the Control group, although they were numerically higher. The SNF content was significantly affected by dietary treatment (*p* = 0.026). The FO and Mix oil groups had higher SNF contents (10.36% and 10.33%, respectively) than the Control group (9.68%; *p* < 0.05), whereas the BSFL oil group showed an intermediate value. Other parameters, including density, TP and lactose, were not statistically altered by dietary treatments (*p* > 0.05), while the mineral salts content was significantly affected by diet (*p* = 0.042), the Mix oil group showing a higher value than the Control group.

### 2.3. Fatty Acid Profile of Transitional and Mature Milk

**Figure 1:**
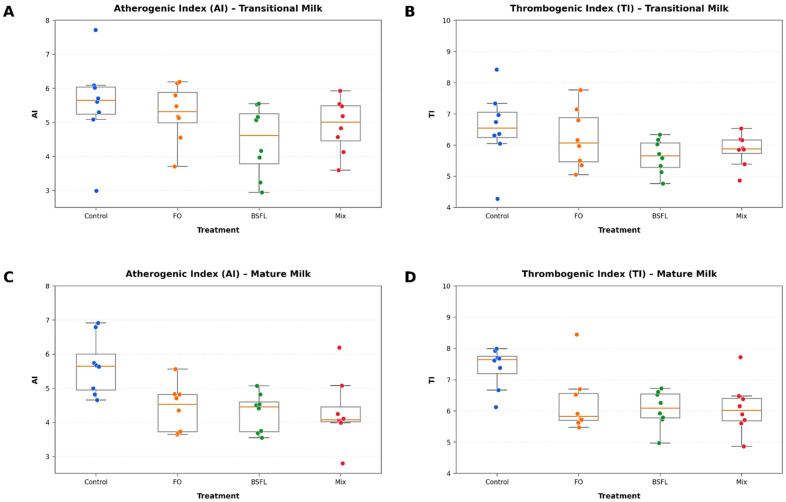
Distribution of the atherogenic index (AI) and thrombogenic index (TI) in transitional and mature sheep milk from ewes receiving different prepartum dietary treatments. The panels show: (**A**) AI in transitional milk, (**B**) TI in transitional milk, (**C**) AI in mature milk, and (**D**) TI in mature milk. Boxes represent the interquartile range, the horizontal orange line indicates the median, whiskers indicate the data range, and individual points represent ewe-level observations. Groups: Control, FO—flaxseed oil, BSFL—black soldier fly larvae oil, Mix oil—2:1 mixture of FO and BSFL oil.

The fatty acid (FA) profile of transitional milk in ewes supplemented with different dietary oils is presented in Table 5. The dietary treatments did not significantly affect the total proportions of saturated fatty acids (SFA), monounsaturated fatty acids (MUFA), and polyunsaturated fatty acids (PUFA) (*p* > 0.05). Similarly, the atherogenic index (AI) and thrombogenic index (TI) remained statistically unchanged in all experimental groups (Figure 1).

Among the individual saturated fatty acids, palmitic acid (C16:0) was the most abundant, ranging from 34.36% to 35.50% of total FAs, followed by stearic acid (C18:0) and myristic acid (C14:0); however, their concentrations were not significantly influenced by oil supplementation (*p* > 0.05). In contrast, dietary treatments significantly altered the proportions of minor saturated FAs, specifically C13:0 (*p* = 0.038) and anteiso-C16:0 (*p* = 0.015). The proportion of C13:0 was significantly lower in the Mix oil group (0.06%) compared to the Control group (0.09%; *p* < 0.05). On the contrary, the inclusion of pure BSFL oil led to a significant increase in the anteiso-C16:0 concentration (0.09%) compared to the flaxseed oil (0.05%) and Mix oil (0.05%) groups (*p* < 0.05), while the Control group exhibited an intermediate value (0.07%). A trend toward a reduction in total medium-chain fatty acids (MCFA) and individual short-chain FAs (C8:0 and C10:0) was observed in the BSFL oil group, though it did not reach the threshold of statistical significance (*p* = 0.078 for C10:0).

Regarding the unsaturated fatty acid fraction, oleic acid (C18:1 *cis*-9) was the predominant MUFA, showing an elevated numerical value in the BSFL oil group (15.00%) compared to the Control (13.16%), though this difference was not statistically significant (*p* > 0.05). No significant dietary effects were detected for major PUFAs, including linoleic acid (C18:2 n-6, *cis*), α-linolenic acid (C18:3 n-3; ALA), and rumenic acid (cis-9, trans-11 CLA) (*p* > 0.05). However, the total PUFA content showed a marginal, nearly significant upward trend (*p* = 0.059) in the groups supplemented with pure oils (FO and BSFL) compared to the Control group. The n-6/n-3 ratio and other structured lipid groups, including odd-chain (OCFA), branched-chain (BCFA), and odd- and branched-chain fatty acids (OBCFA), were stable and did not differ among the treatments (*p* > 0.05).

Table 6 details the fatty acid concentrations, structural lipid categories, and nutritional quality indices recorded during the mature lactation phase. In distinct contrast to the earlier transitional period, the introduction of prepartum dietary oil supplementation affected substantial, statistically clear reconfigurations across the primary lipid fractions, sum parameters, and health-related criteria of the milk.

Total saturated fatty acid (SFA) levels fell markedly under the influence of all oil treatments compared to the Control group (*p* = 0.004). The highest SFA proportion occurred in the Control samples (85.31%), whereas oil inclusion suppressed these values to a range between 82.15% and 82.85% (*p* < 0.05). Looking at specific saturated components, myristic acid (C14:0) reacted significantly to the diets (*p* = 0.018), with the Mix oil group dropping to the lowest level (9.61%) relative to the Control (11.64%; *p* < 0.05), while single oil supplementations yielded intermediate scores. The minor compound anteiso-C16:0 followed a similar pattern (*p* = 0.036), decreasing to 0.05% in the Mix oil group from a baseline of 0.09% in the Control (*p* < 0.05). In contrast, palmitic acid (C16:0) and the sum of medium-chain fatty acids (MCFA) remained steady, showing no statistical variance between cohorts (*p* > 0.05).

All oil treatments increased the total UFA proportion compared with the Control group (*p* = 0.004). The MUFA proportion was higher in the BSFL oil and Mix oil groups than in the Control group, whereas the FO group showed an intermediate value (*p* = 0.006). Oleic acid (C18:1 *cis*-9) was the primary driver behind this change (*p* = 0.004); its share rose from 11.37% in the Control group up to 13.23–13.82% in ewes receiving FO, BSFL or Mix oil (*p* < 0.05). Additionally, total polyunsaturated fatty acid (PUFA) proportion increased following oil delivery (*p* = 0.025), peaking at 3.32% in the pure flaxseed oil group versus 2.63% in the Control (*p* < 0.05). Within the PUFA pool, rumenic acid (*cis*-9, *trans*-11 CLA) showed the most profound response (*p* < 0.001), with a marked increase in the flaxseed oil (0.56%) and BSFL oil (0.50%) groups compared to the Control group (0.32%; *p* < 0.05). Minor shifts were observed for C18:2 isomers (*p* = 0.002), with the BSFL oil group reaching a higher proportion (0.06%) than both the Control and Mix oil groups (0.04%; *p* < 0.05). Both the atherogenic index (AI; *p* = 0.004) and the thrombogenic index (TI; *p* = 0.006) decreased noticeably in all oil-treated groups. The AI fell from 5.65 in the Control group to 4.29–4.42 in the remaining cohorts, while the TI values were brought down from 7.38 to a range of 6.07–6.27 (*p* < 0.05) (Figure 1). In contrast, total fractions of OCFA, BCFA, OBCFA and the n-6/n-3 ratios remained uniform, showing no sensitivity to dietary modifications (*p* > 0.05).

## 3. Discussion

Supplementation of ruminant diets with various oils or oil-rich additives is a well-established nutritional strategy to modify the fatty acid composition of milk and enhance its functional properties [1,3,5,16,18,19,20,21,33,34,35,36]. By altering biohydrogenation pathways, dietary lipids may modify the profile of milk fat to achieve a more desirable balance [36,37,38,39,40,41]. This includes increasing the content of health-beneficial polyunsaturated fatty acids (PUFA), particularly conjugated linoleic acid (*cis*-9, *trans*-11 CLA), while simultaneously reducing the proportion of saturated fatty acids (SFA) with atherogenic potential. Previous studies demonstrate that integrating alternative fat sources, such as Black Soldier Fly-derived products, may affect rumen fermentation and methane production depending on the dose and form of supplementation [42,43]. These metabolic changes not only improve the technological properties of dairy products, but also significantly increase their potential cardiovascular benefits for consumers [2,3,4,5,44,45,46,47,48].

The impact of such dietary interventions on the gross chemical composition of sheep milk is highly dependent on the lactation stage and the specific lipid source. In the present study, prepartum supplementation with flaxseed oil (FO), insect oil (BSFL), or their binary blend exerted no statistically significant effect (*p* > 0.05) on the basic parameters of transitional milk, including fat, solids without fat and total protein. While this lack of initial response contrasts with numerous mid-lactation trials, it may be related to peripartum physiology. During early colostrogenesis, the mobilization of non-esterified fatty acids (NEFAs) from endogenous adipose tissue may be intensified to combat acute negative energy balance [49,50]. Consequently, the contribution of dietary fatty acids to early mammary secretions may have been less apparent against the background of endogenous lipid mobilization. However, this mechanism was not directly assessed in the present study.

Conversely, as lactation progressed and maternal metabolism shifted from tissue mobilization to dietary nutrient extraction, a pronounced carry-over effect emerged. Analysis of mature milk revealed a significantly higher fat content within the BSFL oil group (6.09%) than in the Control group (4.70%; *p* < 0.01), whereas the Mix oil group (5.68%) showed an intermediate value and did not differ significantly from either group. The higher fat content observed in the BSFL oil and Mix oil groups may be associated with differences in the amount and composition of fatty acids supplied by the experimental oils. BSFL oil contained a high proportion of medium-chain fatty acids (MCFAs), predominantly lauric acid (C12:0) [51,52]. The higher mature-milk fat content observed in the BSFL oil group may be associated with the fatty acid composition of the supplement; however, the underlying ruminal mechanism cannot be determined because rumen fermentation parameters were not assessed. This highly concentrated, immediate energy substrate likely alleviated the ewes’ negative energy balance, providing a critical caloric surplus to drive mammary triacylglycerol synthesis.

During the expansion of the lipid fraction, the content of solids not fat (SNF) was significantly affected by dietary treatment, with the FO and Mix oil groups showing a higher value than the Control group (10.36% and 10.33% respectively, vs. 9.68%; *p* = 0.026), whereas the BSFL oil group showed an intermediate value. Lactose levels remained statistically unchanged, while total protein showed only a tendency toward an increase (*p* = 0.078). Mineral salts content was also significantly affected by diet (*p* = 0.042), with the highest value observed in the Mix oil group. These changes may suggest a modest effect of prepartum lipid supplementation on selected non-fat milk components during established lactation; however, further studies including metabolic indicators are needed to confirm the underlying mechanisms.

Beyond the macroscopic composition, the most profound modifications occurred within the structural lipid architecture of mature milk. Significant alterations in the SFA, MUFA, and CLA fractions were observed in mature milk, despite the cessation of oil delivery at lambing. This delayed physiological response may be related to two synchronized mechanisms. First, the prepartum period served as a metabolic priming phase, depositing targeted fatty acids into maternal adipose tissues, which were subsequently released and processed via the mammary Δ9-desaturase system. Second, high-fat diets, particularly those rich in lauric acid, induce robust structural modifications within the ruminal microbiome [40,53]. This creates a microbial carry-over effect that prolongs the postpartum supply of biohydrogenation intermediates into the bloodstream.

As a result, the dietary interventions successfully remodeled the milk fat profile by significantly suppressing total SFAs. This reduction was mainly driven by the decline in myristic acid (C14:0), a recognized risk factor in human cardiovascular disease [47,48]. Mechanistically, the influx of long-chain fatty acids (LCFAs) from the supplements inhibits the mammary gland’s de novo lipogenesis pathway, thereby limiting the endogenous synthesis of short- and medium-chain SFAs. This drop in saturation was counterbalanced by a simultaneous increase in the MUFA pool, driven predominantly by oleic acid (C18:1 *cis*-9). Because dietary fats undergo intensive ruminal biohydrogenation—yielding large amounts of stearic acid (C18:0)—the mammary gland must actively desaturate C18:0 back to C18:1 *cis*-9 to maintain the critical fluidity of the milk fat globule membrane prior to secretion [43,54].

Within the polyunsaturated fraction, the near two-fold enrichment of rumenic acid (*cis*-9, *trans*-11 CLA) in the pure FO (0.56%) and BSFL oil (0.50%) cohorts emerged as a key finding. While the flaxseed oil utilized its abundant linoleic acid (LA, C18:2 n-6, *cis*) as a direct substrate for ruminal isomerization into CLA [22], the pure BSFL oil matched this CLA-stimulating capacity despite its minimal native PUFA content. This phenomenon suggests an insect-specific, lauric acid-driven modulation of Gram-positive ruminal strains, altering biohydrogenation pathways to favor CLA accumulation [40,55]. Interestingly, during the earlier transitional phase, BSFL oil delivery triggered a transient but significant spike in anteiso-C16:0—a branched-chain fatty acid intimately linked to microbial cell membrane adaptations under environmental stress [56,57,58]. This early BCFA fluctuation strongly hints at an initial reconfiguration of the ruminal community in response to the novel insect lipids. The mechanistic interpretation of these findings is limited by the absence of measurements of rumen pH, volatile fatty acids, biohydrogenation intermediates, circulating metabolites, and ruminal microbial composition.

From a nutritional perspective, the significant reduction in both the Atherogenic Index (from 5.65 in the Control group to 4.29–4.42 in the oil-supplemented groups; *p* = 0.004) and the Thrombogenic Index (from 7.38 to 6.07–6.27 respectively; *p* = 0.006) indicates a shift toward a fatty acid profile with lower calculated atherogenic and thrombogenic potential. However, AI and TI are theoretical indices derived from fatty acid composition and do not directly demonstrate cardiovascular effects in consumers. Therefore, the observed reductions should be interpreted as an improvement in the quality of the calculated milk lipids rather than as evidence of compliance with the cardiovascular dietary guidelines.

While the current dataset demonstrates an effective biochemical transformation of mature sheep milk fat, certain methodological boundaries chart the course for future investigations. Because the sampling points within each lactation stage were averaged for each ewe, the analysis reflects treatment effects at the lactation-stage level and does not describe variation among individual sampling days. Although prepartum lipid priming yielded a beneficial carry-over effect during early lactation, extended longitudinal tracking is necessary to determine the lifetime efficacy of this strategy across multiple reproductive cycles. Furthermore, targeted metagenomic profiling is required to definitively elucidate how insect-derived lipids alter the ruminal cellulolytic consortium and the downstream synthesis of functional fatty acids. Finally, tracking the transfer of these bioactive lipids into lamb tissues to evaluate their impact on neonatal immunity and growth performance would fully validate the holistic agricultural value of this sustainable nutritional intervention.

## 4. Materials and Methods

### 4.1. Ethical Procedure

The authors confirmed that all methods were carried out in accordance with the applicable guidelines and regulations. The Animals Ethical Team of Wrocław University of Environmental and Life Sciences approved all of the experimental animals and methodology used in this study. The Local Ethics Committee for Animal Experimentation in Wrocław approved the experiment in its decision: No. 064/2023/P1 of 6 December 2023.

### 4.2. Animals and Experimental Design

The study was carried out at the Research and Teaching Station of the Wrocław University of Environmental and Life Sciences located in Swojczyce (Poland). Thirty-two pregnant Olkuska ewes (3–5 years) were used in the animal study and randomly assigned to four equal groups (*n* = 8 per group): a Control group and three treatment groups supplemented with low-linolenic/high-linoleic flaxseed oil, BSFL oil and a 2:1 mixture of flaxseed and insect oils. The animals were randomly assigned to the experimental groups. The size of the group was based on comparable feeding studies in sheep and practical considerations [59]. A sensitivity analysis showed that, with four groups and eight ewes per group, the design had 80% power to detect a large overall treatment effect (Cohen’s f = 0.63; α = 0.05). Therefore, smaller treatment effects may have remained undetected. All animals were in the late gestation period (pre-parturition stage), aged 3–5 years with an average body weight of 65 ± 5 kg. During the periparturient period, the ewes were housed indoors under standard environmental conditions and had free access to fresh water and a basal diet. Pregnancy was confirmed via transabdominal ultrasonography performed by a veterinarian, and based on these results, the ewes were selected for the study. To ensure homogeneity of the experimental material and minimize the confounding effect of litter size on lambing difficulty, only ewes carrying twin or triplet fetuses were included in the study. Ewes with unconfirmed pregnancy, singletons or more than three fetuses were excluded. Following selection, the animals were assigned to groups using a computer-generated random sequence. To minimize handling-related stress, all animals were managed by a consistent team of trained personnel familiar with the animals. Before the commencement of the study, a mandatory handling and socialization period was conducted to acclimate the animals to experimental procedures and human contact.

No euthanasia was performed in this study as it did not involve terminal procedures. The animals remained under veterinary supervision throughout the experiment. No humane endpoints were reached, and no adverse events or signs of distress were observed that would require medical intervention or premature withdrawal of any animal from the study. After the conclusion of the trial, the animals returned to the home flock.

### 4.3. Diets and Feeding

During the experimental period (21 days prepartum), all sheep were fed a basal diet consisting of meadow hay provided ad libitum and a custom-made concentrate mixture administered at a restricted amount of 0.5 kg per animal twice a day. The animals had free access to fresh drinking water throughout the experimental period. In this study, the experimental diets were supplemented with two distinct lipid sources: cold-pressed flaxseed oil derived from a commercial low-linolenic/high-linoleic oil (LinumVital^®^, Oleofarm, Wrocław, Poland) and insect oil extracted from the larvae of the black soldier fly (*Hermetia illucens*), which was supplied by Grinsect (BSF Systems Kft., Hódmezővásárhely, Hungary). According to the manufacturer’s nutritional declaration, the selected flaxseed oil is atypical, containing a high proportion of omega-6 (approx. 65 g/100 mL) and a substantially reduced omega-3 content (approx. 3 g/100 mL), which was strictly confirmed by our prior GC-MS analysis. In accordance with the manufacturers’ recommendations, all lipid supplements were stored under refrigeration at 7 °C throughout the trial to maintain their quality and prevent oxidation. The oils were administered at a daily dose of 30 g per ewe. To ensure precise dosing and complete ingestion, the assigned oil dose was thoroughly mixed into the daily concentrate allowance and fed individually to each animal. No feed refusals were observed during the trial, confirming the complete intake of the targeted lipid dose. The fatty acid compositions of the administered oils and their mixture were determined via gas chromatography prior to the experimental period to validate the dietary treatment. The detailed fatty acid compositions of the applied oils are presented in Table 3.

### 4.4. Sample Protocol

To evaluate the parameters of transitional and mature milk, a systematic sampling protocol was implemented. To maintain hygiene and minimize sample contamination, disposable gloves were worn by the personnel at each sampling time point. The first few streams of secretion were discarded to ensure that the samples reflected the internal composition of the mammary gland. Approximately 30–50 mL of secretion was hand-milked from both teats into sterile plastic tubes. For the purposes of the present study, samples collected at 24 h and on day 3 postpartum were operationally classified as transitional milk, whereas samples collected on days 7 and 21 postpartum were classified as mature milk. This classification was based on previous studies describing rapid changes in ovine milk composition during the first postpartum days and the transition toward a more stable milk composition by approximately the end of the first week [32,60]. The four sampling time points were selected to represent the early postpartum transition (24 h and day 3), the mature milk period (day 7), and a later stage of early lactation (day 21), allowing the evaluation of potential post-supplementation effects after supplementation had ceased. Samples were obtained from each ewe across the four groups. At each stage of lactation, two milk samples were collected from each of the eight ewes, resulting in 16 sample collections per treatment group. Because repeated samples obtained from the same ewe were not statistically independent, they were not treated as separate experimental units. Immediately after collection, each sample underwent an initial qualitative assessment using a digital refractometer (Milwaukee Instruments Inc., Rocky Mount, NC, USA). Upon completion of these measurements, the biological material was promptly chilled and subsequently stored at −20 °C to ensure preservation for further laboratory analyses.

**Figure 2:**
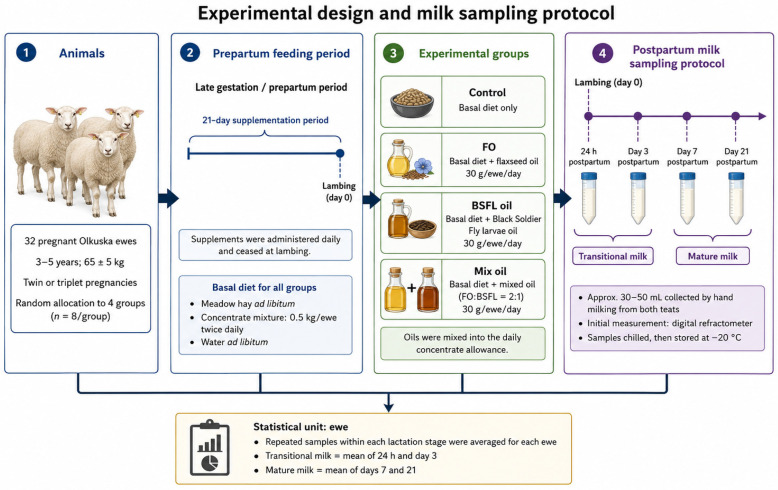
Schematic overview of the experimental design and the postpartum milk sampling protocol.

Blinding was applied during laboratory analyses and statistical processing. Although the personnel administering the supplements were aware of the group allocation due to the nature of the interventions, laboratory staff and the statistician were blinded to the treatment codes until the final data analysis was complete. This approach was taken to reduce potential bias during sample processing. The experimental design and postpartum milk sampling protocol are summarized in Figure 2.

### 4.5. Laboratory Analyses

#### 4.5.1. Basal Diet Composition

The proximate composition of the basal diets and experimental lipid supplements was evaluated in triplicate. Dry matter (DM, procedure III A), crude ash (procedure III M), crude protein (procedure III C), and crude fat (ether extract, procedure III H) were determined according to the official protocols of the Commission Regulation (EC) No 152/2009 [61]. The crude protein analysis, based on the Kjeldahl method, was further validated according PN-EN ISO 5983-1 standard [62]. To quantify the fibrous fractions, the filter bag technique was employed; crude fiber was measured on an ANKOM Delta automated analyzer (ANKOM Technology, Macedon, NY, USA) following the AOCS Ba 6a-05 procedure and the PN-ISO 6541:1981 standard [63,64]. The neutral detergent fiber (NDF) and acid detergent fiber (ADF) fractions were analyzed using the same ANKOM Delta system according to the manufacturer’s guidelines and the AOCS Ba 6a-05 protocols [63]. Mineral concentrations were determined using standardized spectroscopic techniques: calcium (Ca) content was quantified via atomic absorption spectrometry according to PN-EN ISO 6869 and PN-EN 14084 [65,66], whereas total phosphorus (P) was measured photometrically in compliance with the PN-ISO 6491 guidelines [67].

#### 4.5.2. Fatty Acids

To determine the fatty acid profile of the basal diet, the hay and concentrate samples were homogenized individually by grinding and mixing. A 200 mg portion of each sample was weighed and spiked with 0.05 mg of heptadecanoic acid (C17:0), used as an internal standard. A mixture of chloroform and methanol was added in a 2:1 ratio to the sample, shaken and centrifuged. The layer containing the Folch liquid was collected and evaporated. Two milliliters of a 1 M potassium hydroxide solution in methanol were added to a round-bottom flask. The mixture was then heated in a heating basket (80 °C) for three minutes with the lid on. Then, 1.5 mL of BF_3_ solution was added, after which the mixture was heated again (80 °C) for three minutes. A total of 1 mL of distilled water and 1 mL of aqueous NaCl solution were used for cooling. A total of 5 mL of cyclohexane was added, after which the samples were shaken vigorously. The mixture was transferred to 15 mL Falcon conical tubes and mixed in a vortex mixer for a further 30 s. It was then centrifuged for four minutes in a table centrifuge at 14,500× *g*. The upper cyclohexane phase was collected and filtered through a cotton and celite layer before being transferred to an evaporation dish with a pipette. The liquid phase was then completely evaporated to dryness, and 500 µL of cyclohexane was added. The vessel was thoroughly rinsed with it and a sample was transferred to the glass vials.

For fatty acid analysis, 10 mL of each sample of transitional and mature milk was transferred into 15 mL conical centrifuge tubes and centrifuged for 15 min at 14,500× *g* in a tabletop centrifuge. The separated fat fraction was collected and transferred into 2 mL microcentrifuge tubes. Subsequently, 30 mg of the extracted fat was weighed in triplicate into 20 mL glass vials for extraction. To the samples, 2 mL of 1 M KOH in methanol and 0.5 mg of an internal standard, nonadecanoic acid (C19:0) were added. The prepared mixture was heated at 80 °C for 10 min on a magnetic hotplate stirrer. Next, 1.5 mL of BF_3_ was added, and the samples were heated at 80 °C in closed vials for an additional 3 min. For rapid cooling, 1 mL of distilled water and 1 mL of aqueous NaCl solution were added. Cyclohexane was then introduced and the samples were vigorously shaken for 30 s. The mixture was transferred into 15 mL conical Falcon tubes, vortexed for another 30 s, and centrifuged for 4 min in a benchtop centrifuge (14,000× *g*). The upper cyclohexane phase was collected, filtered through a Celite bed, and transferred by pipette into an evaporation vessel, where the liquid phase was completely evaporated to dryness. Following total evaporation, 1 mL of cyclohexane was added to thoroughly rinse the bottom of the vessel, and then the whole mixture was transferred into a chromatography vial and analyzed. Each sample was analyzed in triplicate.

Fatty acid methyl esters (FAMEs) from meadow hay, concentrate mixture and milk fat were analyzed using the Shimadzu GC-MS QP 2020 system (Shimadzu, Kyoto, Japan). Chromatographic separation was performed on a Zebron ZB-FAME capillary column (60 m × 0.25 mm × 0.20 μm; Phenomenex, Torrance, CA, USA). The injection port temperature was set to 280 °C. The split ratio was set to 1:80 for milk fat samples. The oven temperature program was initiated at 80 °C (hold for 2 min), followed by a ramp of 3 °C min^−1^ to 180 °C, then 8 °C min^−1^ to 240 °C with a final hold of 4 min. Helium was used as the carrier gas at a constant column flow of 1.80 mL min^−1^. The ion source and interface temperatures were maintained at 220 °C and 250 °C, respectively.

Fatty acids were identified by comparing their mass spectra with the NIST 20 mass spectral library and by matching their retention times with a commercial standard mixture (Supelco 37 Component FAME Mix, Sigma-Aldrich, St. Louis, MO, USA). The concentration of each fatty acid was expressed as a percentage of total FAs. Subsequently, the FA groups and their nutritional properties were calculated.

#### 4.5.3. Milk Physicochemical Analysis

The fat, solids-not-fat, density, total protein, lactose, and mineral salts contents of transitional and mature milk were determined using a Farm Eco milk analyzer (Milkotester Ltd., Stara Zagora, Bulgaria). Before analysis, the milk samples were heated to 40 °C in a water bath and gently mixed to ensure homogenization, in accordance with the manufacturer’s instructions.

### 4.6. Statistical Analysis

Statistical analysis was performed using Statistica software (version 13, TIBCO Software Inc., Palo Alto, CA, USA). The ewe was considered the experimental unit. Prior to statistical analysis, the results of three technical chromatographic replicates obtained for each biological sample were averaged to ensure precision. To account for within-animal dependence and avoid pseudoreplication, values obtained at 24 h and on day 3 postpartum were averaged within each ewe to generate one transitional-milk observation, while values obtained on days 7 and 21 postpartum were averaged to generate one mature-milk observation. Thus, each lactation stage comprised eight independent ewe-level observations per treatment group. Transitional and mature milk were analyzed separately. This stage-level approach was used to compare dietary treatments within each lactation stage rather than to evaluate changes between individual sampling days. To evaluate the nutritional and health-promoting properties of sheep milk fat, specific fatty acid groups and lipid indices were calculated on the individual fatty acid profiles. The groups of fatty acids were defined and calculated as follows:

SFA = Σ (C8:0 + C10:0 + C12:0 + C13:0 + C14:0 + iso-C15:0 + anteiso-C15:0 + C15:0 + iso-C16:0 + C16:0 + anteiso-C16:0 + iso-C17:0 + anteiso-C17:0 + C17:0 + iso-C18:0 + C18:0 + C22:0),(1)

MUFA = Σ (C16:1 *cis*-9 + C17:1 *cis*-10 + C18:1 *cis*-9 + C18:1 n-7 + C18:1 isomers + C20:1 n-9), (2)

PUFA = Σ (C18:2 n-6 + C18:2 isomers + C18:3 n-3 + *cis*-9, *trans*-11 CLA + CLA isomers + C20:4 n-6 + C20:5 n-3 + C22:5 n-3 + C22:6 n-3), (3)

UFA = MUFA + PUFA,(4)

MCFA = Σ (C8:0 + C10:0 + C12:0),(5)

LCFA = Σ (C13:0 + C14:0 + iso-C15:0 + anteiso-C15:0 + C15:0 + iso-C16:0 + C16:0 + anteiso-C16:0 + C16:1 *cis*-9 + iso-C17:0 + anteiso-C17:0 + C17:0 + C17:1 cis-10 + iso-C18:0 + C18:0 + C18:1 *cis*-9 + C18:1 n-7 + C18:1 isomers + C18:2 n-6 + C18:2 isomers + C18:3 n-3 + *cis*-9, *trans*-11 CLA + CLA isomers + C20:1 n-9 + C20:4 n- 6 + C20:5 n-3 + C22:0 + C22:5 n-3 + C22:6 n-3),(6)

BCFA = Σ (iso-C15:0 + anteiso-C15:0 + iso-C16:0 + anteiso-C16:0 + iso-C17:0 + anteiso-C17:0 + iso-C18:0),(7)

OCFA = Σ (C13:0 + iso-C15:0 + anteiso-C15:0 + C15:0 + iso-C17:0 + anteiso-C17:0 + C17:0 + C17:1 *cis*-10), (8)

OBCFA = BCFA + OCFA (excluding double-counted branched-chain fractions),(9)

n-6 PUFA = Σ (C18:2 n-6 + C20:4 n-6),(10)

n-3 PUFA = Σ (C18:3 n-3 + C20:5 n-3 + C22:5 n-3 + C22:6 n-3).(11)

The Atherogenic Index (AI) and the Thrombogenic Index (TI), which illustrate the potential impact of milk fat consumption on cardiovascular health, were determined using the classic equations proposed by Ulbricht and Southgate [68]:

(12) $$AI\;=\;\frac{\left(C12:0\;+\;\left(4\;\times \;C14:0\right)\;+\;C16:0\right)}{\left(MUFA\;+\;\Sigma n\;-\;6\;+\;\Sigma n-3\right)}$$

(13) $$TI=\frac{\left(C14:0+C16:0+C18:0\right)}{\left(\left(0.5\times MUFA\right)+\left(0.5\times \Sigma n-6\right)+\left(3\times \Sigma n-3\right)+\left(\frac{n-3}{n-6}\right)\right)}$$

The normal distribution of the data within dietary treatments was evaluated using the Shapiro–Wilk test, and the homogeneity of variances was verified using Levene’s test. Depending on the fulfillment of parametric assumptions, one of three statistical pathways was applied for each fatty acid:

For variables that exhibit a normal distribution and homogeneous variances, a standard one-way analysis of variance (ANOVA) was performed, followed by Tukey’s post hoc test. For these variables, the pooled standard error of the mean $SEM=\sqrt{MSE∕n}$ was reported.

For variables with a normal distribution but heterogeneous variances (*p* < 0.05) in Levene’s test, Welch’s F test was conducted to determine the overall effect of treatment, and the highest standard error between the groups (Max SEM) from descriptive statistics was reported.

For variables displaying a non-parametric distribution (*p* < 0.05) in the Shapiro–Wilk test, the Kruskal–Wallis test was employed, followed by the non-parametric multiple comparisons test for mean ranks. To maintain a uniform table structure, results are presented as arithmetic means and the maximum standard error (Max SEM) is reported. Individual ewe-level values were inspected using boxplots and Q–Q plots. Potential outliers were identified using the 1.5 × IQR rule for graphical assessment; no observations were excluded unless a documented analytical or data entry error was identified.

Data styling and dispersion metrics were adjusted according to the nature of the evaluated matrices. For the characterization of the experimental oil supplements (Table 3), results are presented as Mean ± SD (standard deviation) to accurately reflect the internal analytical variance of the raw materials. For Table 4, Table 5 and Table 6, data are presented as arithmetic means accompanied by either the pooled SEM for variables analyzed by standard one-way ANOVA or the highest group SEM for variables analyzed using Welch’s ANOVA or the Kruskal–Wallis test, as specified in the table footnotes. The use of SEM in these cohorts was selected as the standard biometric approach to illustrate the precision of population mean estimates and the overall stability of the experimental treatment effects across the biological replicates (*n* = 8 per group).

## 5. Conclusions

Prepartum supplementation with flaxseed oil, Black Soldier Fly larvae oil, and their blend modified the chemical composition and fatty acid profile of mature sheep milk. In transitional milk, no significant treatment effects were observed for the gross composition, major fatty acid classes, AI, or TI, although differences were detected for selected minor fatty acids. The BSFL oil supplementation significantly increased mature milk fat content, while all supplemented groups showed lower AI and TI indices compared with the Control group. Furthermore, FO and BSFL oil supplementation increased CLA concentration, while combined treatment with FO and BSFL oil significantly reduced the proportion of atherogenic myristic acid (C14:0). These findings indicate that prepartum supplementation with plant- and insect-derived lipid sources may represent a useful nutritional strategy for improving selected lipid quality indices of mature sheep milk and may contribute to enhancing its nutritional value for consumers. Consequently, this approach may support the development of sheep milk with desirable characteristics for functional food production.

However, the results should be interpreted in light of the limitations of the study, including the relatively small number of animals, the prepartum supplementation period, and the lack of metabolic measurements that would allow a more comprehensive assessment of the underlying physiological mechanisms. Therefore, further studies involving larger populations, longer supplementation periods, and evaluation of maternal and offspring metabolic responses are required to confirm these findings and determine their practical significance.

## Figures and Tables

**Table 1:** Chemical composition and macromineral content of the basal diet components.

Chemical Composition	Meadow Hay	Concentrate Mixture ^1^
Dry matter [%]	92.83	90.62
% of Dry Matter (DM)		
Ash	6.40	8.25
Crude protein	8.98	16.96
Crude fiber	32.34	11.51
Fat	1.03	9.61
NDF	59.85	33.79
ADF	23.23	10.32
Minerals [g/kg DM]		
Ca	6.47	21.89
P	2.40	5.71
Ca:P	2.70	3.84

^1^ Concentrate mixture ingredients: 50% oats, 30% triticale, 10% soybean meal, 5% maize and 5% mineral-vitamin premix (Polfamix); DM—dry matter; NDF—neutral detergent fiber; ADF—acid detergent fiber; Ca—calcium; P—phosphorus.

**Table 2:** Fatty acid composition of basal diet components [% on the total of FAs].

	Meadow Hay	Concentrate Mixture ^1^
C14:0	1.55	0.23
C16:0	38.29	17.25
C16:1 *cis*-9	1.87	0.12
C18:0	7.48	4.17
C18:1 *cis*-9	4.50	27.02
C18:2 n-6, *cis*	19.44	44.60
C18:3 n-3	17.51	5.03
C20:0	1.73	0.35
C20:1 n-9	*ND*	0.46
C22:0	2.41	0.41
C24:0	5.20	0.36
SFA	56.66	23.23
MUFA	6.37	27.60
PUFA	36.95	49.63
UFA	43.32	77.23
n-6	19.44	44.60
n-3	17.51	5.03
n-6/n-3	1.11	8.87
n-3/n-6	0.90	0.11

Data are expressed as raw analytical means of *n* = 3 replicates; *ND*—not detected; ^1^ Concentrate mixture ingredients: 50% oats, 30% triticale, 10% soybean meal, 5% maize and 5% mineral-vitamin premix (Polfamix); SFA—saturated fatty acids (sum of C14:0, C16:0, C18:0, C20:0, C22:0, and C24:0); MUFA—monounsaturated fatty acids (sum of C16:1, C18:1 and C20:1 n-9); PUFA—polyunsaturated fatty acids (sum of C18:2 and C18:3); UFA—unsaturated fatty acids (MUFA + PUFA).

**Table 3:** Fatty acid composition of low-linolenic flaxseed oil, BSFL oil, and their 2:1 mixture [% of total FAs].

	FO	BSFL Oil	Mix Oil ^1^
C8:0	*ND*	0.01 ± 0.01	*ND*
C10:0	0.01 ± 0.01	1.51 ± 0.03	0.62 ± 0.00
C12:0	0.18 ± 0.22	51.93 ± 0.44	25.07 ± 1.60
C14:0	0.15 ± 0.06	11.72 ± 0.07	4.91 ± 0.02
iso-C15:0	*ND*	0.06 ± 0.00	0.03 ± 0.00
anteiso-C15:0	*ND*	0.02 ± 0.00	0.01 ± 0.00
C15:0	0.07 ± 0.01	0.08 ± 0.01	0.04 ± 0.02
iso-C16:0	*ND*	0.01 ± 0.00	0.01 ± 0.00
anteiso-C16:0	0.02 ± 0.00	0.14 ± 0.00	0.06 ± 0.00
C16:0	16.26 ± 0.19	19.38 ± 0.22	16.48 ± 0.60
C16:1 *cis*-9	0.09 ± 0.00	1.17 ± 0.01	0.51 ± 0.01
iso-C17:0	*ND*	0.01 ± 0.00	0.01 ± 0.00
anteiso-C17:0	*ND*	0.02 ± 0.00	0.01 ± 0.00
C17:0	0.19 ± 0.00	0.07 ± 0.00	0.11 ± 0.06
C17:1 *cis*-10	0.04 ± 0.00	0.02 ± 0.01	0.02 ± 0.00
iso-C18:0	*ND*	*ND*	*ND*
C18:0	11.30 ± 0.21	2.45 ± 0.08	6.18 ± 0.05
C18:1 *cis*-9	13.60 ± 0.04	5.25 ± 0.01	8.89 ± 0.07
C18:1 n-7	*ND*	*ND*	0.01 ± 0.01
C18:2 n-6, *cis*	54.66 ± 0.30	5.34 ± 0.02	34.95 ± 0.33
C18:2 n-6, *trans*	0.02 ± 0.01	0.02 ± 0.00	0.02 ± 0.00
C18:2 *cis*-9, *trans*-11 (CLA)	0.01 ± 0.00	0.02 ± 0.00	0.01 ± 0.01
C18:3 n-3	2.96 ± 0.02	0.59 ± 0.00	1.75 ± 0.04
C20:0	0.01 ± 0.00	0.12 ± 0.00	0.04 ± 0.00
C20:1 n-9	0.03 ± 0.01	0.01 ± 0.00	0.01 ± 0.01
C20:2 *cis*-11, *cis*-14	0.03 ± 0.01	0.01 ± 0.00	0.02 ± 0.00
C21:0	0.01 ± 0.00	*ND*	0.01 ± 0.00
C20:4 n-6	*ND*	*ND*	0.01 ± 0.00
C22:0	0.34 ± 0.00	0.02 ± 0.01	0.17 ± 0.00
C20:5 n-3	0.01 ± 0.00	0.02 ± 0.01	0.01 ± 0.00
SFA	28.55 ± 0.28	87.56 ± 0.03	53.75 ± 0.42
MUFA	13.76 ± 0.04	6.45 ± 0.01	9.45 ± 0.09
PUFA	57.69 ± 0.32	5.99 ± 0.03	36.80 ± 0.35
n-3	2.97 ± 0.02	0.60 ± 0.00	1.75 ± 0.04
n-6	54.68 ± 0.30	5.36 ± 0.03	35.02 ± 0.35
UFA	71.45 ± 0.28	12.44 ± 0.03	46.25 ± 0.42
UFA/SFA	2.50 ± 0.03	0.14 ± 0.00	0.86 ± 0.01
n-6/n-3	18.40 ± 0.03	8.96 ± 0.06	20.00 ± 0.47
n-3/n-6	0.05 ± 0.00	0.11 ± 0.00	0.05 ± 0.00
AI	0.24 ± 0.00	9.50 ± 0.02	1.32 ± 0.02
TI	0.64 ± 0.10	4.29 ± 0.06	1.00 ± 0.03

^1^ Mix oil is a 2:1 mixture of FO and BSFL oil; *ND*—not detected; Data are expressed as mean ± standard deviation (SD) of *n* = 3 replicates; FAs—fatty acids; FO—flaxseed oil; BSFL—black soldier fly larvae oil (*Hermetia illucens*); SFA—saturated fatty acids; MUFA—monounsaturated fatty acids; PUFA—polyunsaturated fatty acids; UFA—unsaturated fatty acids; CLA—conjugated linoleic acid; n-3—omega-3 fatty acids; n-6—omega-6 fatty acids; AI—atherogenic index; TI—thrombogenic index.

**Table 4:** Effect of dietary oil supplementation on the chemical composition of sheep transitional milk and mature milk.

	Control	FO	BSFL Oil	Mix Oil ^1^	SEM	*p*-Value
Transitional milk						
Fat [%]	7.42	7.32	7.14	6.66	0.380	0.515
SNF [%]	11.44	11.35	11.06	11.12	0.332	0.861
Density [kg·m^−3^]	1036.94	1036.66	1036.03	1036.40	1.268	0.964
TP [%]	4.17	4.03	4.03	4.05	0.119	0.860
Lactose [%]	6.29	6.25	6.09	6.12	0.183	0.858
Mineral salts [%]	0.94	0.94	0.91	0.91	0.030	0.804
Mature milk						
Fat [%]	4.70 ^b^	5.55 ^ab^	6.09 ^a^	5.68 ^ab^	0.285	0.008
SNF [%]	9.68 ^b^	10.36 ^a^	10.08 ^ab^	10.33 ^a^	0.170	0.026
Density [kg·m^−3^]	1032.60	1033.29	1034.16	1034.25	0.854	0.486
TP [%]	3.54	3.81	3.88	3.77	0.098	0.078
Lactose [%]	5.37	5.60	5.55	5.68	0.097	0.153
Mineral salts [%]	0.79 ^b^	0.82 ^ab^	0.82 ^ab^	0.84 ^a^	0.014	0.042

^1^ Mix oil is a 2:1 mixture of FO and BSFL oil. Milk values were expressed as arithmetic means of transitional (24 h and day 3) and mature (days 7 and 21) samples. The ewe was the experimental unit (*n* = 8 per treatment group). SEM: pooled standard error of the mean (*n* = 8 ewes per group). FO—flaxseed oil; BSFL—black soldier fly larvae oil (*Hermetia illucens*); SNF: solids-not-fat; TP: total protein. *p*-Value stems from one-way ANOVA performed on log-transformed data (where applicable). ^a,b^ Means within a row with different superscripts differ significantly (*p* < 0.05) based on Tukey’s post hoc test.

**Table 5:** Fatty acid profile of transitional milk in ewes supplemented with different oils [% of total FAs].

Transitional Milk	Treatments				*SEM*	*p*-Value
	Control	FO	BSFL Oil	Mix Oil ^1^		
C8:0	2.67	2.61	2.06	2.77	0.288	0.321
C10:0	7.81	8.15	6.20	8.16	1.060	0.078
C12:0	4.59	4.81	4.34	4.53	0.295	0.740
C13:0	0.09 ^b^	0.07 ^ab^	0.07 ^ab^	0.06 ^a^	0.007	0.038
C14:0	12.87	12.65	11.43	12.07	0.632	0.389
iso-C15:0	0.25	0.25	0.25	0.25	0.015	0.987
anteiso-C15:0	0.24	0.25	0.23	0.24	0.016	0.671
C15:0	1.01	0.98	1.08	1.06	0.056	0.550
iso-C16:0	0.20	0.19	0.19	0.19	0.017	0.926
C16:0	34.85	34.36	35.50	34.58	0.717	0.704
anteiso-C16:0	0.07 ^ab^	0.05 ^a^	0.09 ^b^	0.05 ^a^	0.010	0.015
C16:1 *cis*-9	0.34	0.37	0.43	0.38	0.026	0.173
iso-C17:0	0.48	0.47	0.52	0.48	0.034	0.692
anteiso-C17:0	0.43	0.43	0.43	0.45	0.038	0.964
C17:0	0.60	0.57	0.59	0.54	0.055	0.612
C17:1 *cis*-10	0.16	0.14	0.20	0.18	0.023	0.398
iso-C18:0	0.11	0.09	0.13	0.11	0.018	0.420
C18:0	16.55	16.60	17.12	16.26	1.089	0.955
C18:1 *cis*-9	13.16	12.98	15.00	13.85	0.822	0.313
C18:1 n-7	0.28	0.23	0.26	0.24	0.028	0.697
C18:1 isomers	0.10	0.11	0.13	0.10	0.014	0.607
C18:2 n-6, *cis*	1.73	1.95	2.04	1.81	0.104	0.183
C18:2 isomers	0.04	0.04	0.06	0.05	0.005	0.178
C18:3 n-3 (ALA)	0.39	0.48	0.48	0.50	0.036	0.164
C18:2 *cis*-9, *trans*-11 (CLA)	0.48	0.67	0.70	0.60	0.066	0.112
C20:1 n-9	0.03	0.03	0.04	0.03	0.003	0.253
CLA isomers	0.07	0.07	0.07	0.07	0.002	0.660
C22:0	0.11	0.10	0.11	0.11	0.005	0.528
C20:4 n-6 (ARA)	0.06	0.06	0.05	0.05	0.004	0.215
C20:5 n-3	0.05	0.06	0.05	0.06	0.004	0.660
C22:5 n-3	0.11	0.12	0.11	0.11	0.007	0.916
C22:6 n-3	0.06	0.06	0.05	0.05	0.004	0.166
MCFA	15.07	15.56	12.61	15.46	1.291	0.340
LCFA	84.94	84.43	87.39	84.54	1.292	0.341
SFA	82.94	82.63	80.35	81.92	0.971	0.260
MUFA	14.07	13.87	16.04	14.78	0.871	0.301
PUFA	3.00	3.50	3.60	3.30	0.158	0.059
UFA	17.07	17.36	19.64	18.08	0.971	0.262
OCFA	3.26	3.16	3.37	3.26	0.115	0.641
BCFA	1.79	1.74	1.85	1.78	0.100	0.900
OBCFA	3.65	3.50	3.78	3.61	0.136	0.535
n-6 PUFA	1.79	2.00	2.09	1.86	0.106	0.216
n-3 PUFA	0.61	0.71	0.69	0.72	0.045	0.366
n-6/n-3	3.05	2.94	3.02	2.63	0.210	0.491
n-3/n-6	0.35	0.36	0.34	0.39	0.025	0.563

^1^ Mix oil is a 2:1 mixture of FO and BSFL oil. Values represent ewe-level means calculated from samples collected at 24 h and on day 3 postpartum. The ewe was the experimental unit (*n* = 8 per treatment group). ^a,b^ Different superscript letters within the same row indicate statistically significant differences between treatment groups at *p* < 0.05. SEM—pooled standard error of the mean (or the highest standard error among groups where Welch’s ANOVA or Kruskal–Wallis test was applied). FO—flaxseed oil; BSFL oil—black soldier fly larvae oil (*Hermetia illucens*); FAs—fatty acids; ALA—α-linolenic acid; ARA—arachidonic acid; CLA—conjugated linoleic acid; SFA—sum of individual saturated fatty acids; UFA—sum of individual unsaturated fatty acids; MUFA—sum of individual monounsaturated fatty acids; PUFA—sum of individual polyunsaturated fatty acids; OCFA—odd-chain fatty acids; BCFA—branched-chain fatty acids (sum of iso- and anteiso-FAs); OBCFA—odd- and branched-chain fatty acids (sum of odd-, iso- and anteiso-FAs); MCFA—medium-chain fatty acids (sum of individual FAs from C8:0 to C12:0); LCFA—long-chain fatty acids (sum of individual FAs from C13:0 to C22:6 n-3); n-3 PUFA and n-6 PUFA—sums of individual n-3 and n-6 polyunsaturated fatty acids, respectively.

**Table 6:** Fatty acid profile of mature milk in ewes supplemented with different oils [% of total FAs].

Mature Milk	Treatments				*SEM*	*p*-Value
	Control	FO	BSFL Oil	Mix Oil ^1^		
C8:0	2.89	2.60	2.39	3.40	0.432	0.177
C10:0	8.88	7.92	7.27	9.15	0.724	0.254
C12:0	4.92	4.21	4.17	4.37	0.275	0.217
C13:0	0.12	0.10	0.11	0.10	0.013	0.652
C14:0	11.64 ^b^	9.91 ^ab^	10.08 ^ab^	9.61 ^a^	0.455	0.018
iso-C15:0	0.28	0.29	0.28	0.26	0.015	0.554
anteiso-C15:0	0.35	0.36	0.34	0.34	0.020	0.844
C15:0	1.12	1.18	1.19	1.03	0.050	0.112
iso-C16:0	0.28	0.28	0.23	0.27	0.021	0.225
C16:0	30.87	31.32	31.19	29.45	0.574	0.108
anteiso-C16:0	0.09 ^b^	0.06 ^ab^	0.08 ^ab^	0.05 ^a^	0.009	0.036
C16:1 *cis*-9	0.22	0.25	0.27	0.24	0.014	0.081
iso-C17:0	0.46	0.54	0.56	0.52	0.029	0.097
anteiso-C17:0	0.42	0.44	0.46	0.46	0.031	0.673
C17:0	0.09	0.09	0.09	0.08	0.002	0.108
C17:1 *cis*-10	0.11	0.13	0.16	0.14	0.017	0.199
*iso*-C18:0	0.10	0.09	0.13	0.12	0.021	0.375
C18:0	22.74	23.29	23.47	23.54	1.008	0.942
C18:1 *cis*-9	11.37 ^b^	13.23 ^a^	13.82 ^a^	13.52 ^a^	0.462	0.004
C18:1 n-7	0.27	0.19	0.24	0.20	0.026	0.086
C18:1 isomers	0.06	0.09	0.08	0.07	0.007	0.060
C18:2 n-6, *cis*	1.55	1.79	1.80	1.63	0.116	0.352
C18:2 isomers	0.04 ^a^	0.04 ^ab^	0.06 ^b^	0.04 ^a^	0.003	0.002
C18:3 n-3 (ALA)	0.37	0.52	0.52	0.52	0.047	0.080
C18:2 *cis*-9, *trans*-11 (CLA)	0.32 ^b^	0.56 ^a^	0.50 ^a^	0.44 ^ab^	0.034	<0.001
C20:1 n-9	0.02	0.03	0.03	0.03	0.003	0.133
CLA isomers	0.07	0.10	0.09	0.08	0.011	0.058
C22:0	0.09	0.09	0.09	0.08	0.005	0.332
C20:4 n-6 (ARA)	0.09	0.10	0.08	0.08	0.008	0.384
C20:5 n-3	0.04	0.05	0.05	0.05	0.005	0.079
C22:5 n-3	0.10	0.10	0.10	0.09	0.008	0.144
C22:6 n-3	0.05	0.04	0.04	0.03	0.004	0.161
MCFA	16.68	14.72	13.83	16.92	1.193	0.214
LCFA	83.32	85.28	86.17	83.08	1.193	0.214
SFA	85.31 ^b^	82.77 ^a^	82.15 ^a^	82.85 ^a^	0.589	0.004
MUFA	12.07 ^b^	13.92 ^ab^	14.61 ^a^	14.19 ^a^	0.494	0.006
PUFA	2.63 ^a^	3.32 ^b^	3.24 ^ab^	2.96 ^ab^	0.164	0.025
UFA	14.69 ^b^	17.23 ^a^	17.85 ^a^	17.15 ^a^	0.589	0.004
OCFA	2.94	3.13	3.21	2.94	0.123	0.325
BCFA	1.97	2.06	2.08	2.02	0.100	0.848
OBCFA	3.40	3.57	3.64	3.38	0.142	0.506
n-6 PUFA	1.64	1.89	1.89	1.71	0.117	0.343
n-3 PUFA	0.56	0.72	0.71	0.68	0.051	0.131
n-6/n-3	2.94	2.77	2.71	2.53	0.194	0.530
n-3/n-6	0.35	0.38	0.39	0.40	0.027	0.568

^1^ Mix oil is a 2:1 mixture of FO and BSFL oil. Values represent ewe-level means calculated from samples collected on days 7 and 21 postpartum. The ewe was the experimental unit (*n* = 8 per treatment group). ^a,b^ Different superscript letters within the same row indicate statistically significant differences between treatment groups at *p* < 0.05. SEM—pooled standard error of the mean (or the highest standard error among groups where Welch’s ANOVA or Kruskal–Wallis test was applied). FO—flaxseed oil; BSFL oil—black soldier fly larvae oil (*Hermetia illucens*); FAs—fatty acids; ALA—α-linolenic acid; ARA—arachidonic acid; CLA—conjugated linoleic acid; SFA—sum of individual saturated fatty acids; UFA—sum of individual unsaturated fatty acids; MUFA—sum of individual monounsaturated fatty acids; PUFA—sum of individual polyunsaturated fatty acids; OCFA—odd-chain fatty acids; BCFA—branched-chain fatty acids (sum of iso- and anteiso-FAs); OBCFA—odd- and branched-chain fatty acids (sum of odd-, iso- and anteiso-FAs); MCFA—medium-chain fatty acids (sum of individual FAs from C8:0 to C12:0); LCFA—long-chain fatty acids (sum of individual FAs from C13:0 to C22:6 n-3); n-3 PUFA and n-6 PUFA—sums of individual n-3 and n-6 polyunsaturated fatty acids, respectively.

## Data Availability

The datasets used and analyzed during the current study are available from the corresponding authors on reasonable request.

## Supplementary Materials

The following supporting information can be downloaded at: https://www.mdpi.com/article/10.3390/molecules31152628/s1, Figure S1: Ewe-level physicochemical composition of transitional and mature sheep milk.

## Abbreviations

GC-MS	gas chromatography–mass spectrometry
DM	Dry matter
NDF	neutral detergent fiber
ADF	acid detergent fiber
SNF	solids-not-fat
TP	total protein
NEFA	non-esterified fatty acid
FO	flaxseed oil
BSFL oil	black soldier fly larvae oil
FA	fatty acid
LA	linoleic acid
ALA	α-linolenic acid
ARA	arachidonic acid
CLA	conjugated linoleic acid
SFA	saturated fatty acids
UFA	unsaturated fatty acids
MUFA	monounsaturated fatty acids
PUFA	polyunsaturated fatty acids
OCFA	odd-chain fatty acids
BCFA	branched-chain fatty acids
OBCFA	odd- and branched-chain fatty acids
MCFA	medium-chain fatty acids
LCFA	long-chain fatty acids
TI	thrombogenic index
AI	atherogenic index
