# Supplementary material for: Effects of Prepartum Supplementation with Flaxseed and Black Soldier Fly Larvae Oils on Fatty Acid Composition and Lipid Quality Indices in Sheep Milk
Source: Molecules. 2026 Jul 28;31(15):2628. doi: 10.3390/molecules31152628 (PMC13467788; doi:10.3390/molecules31152628)
Supplement: Supplementary file 1 [file molecules-31-02628-s001.zip › molecules-4376133-supplementary.pdf]

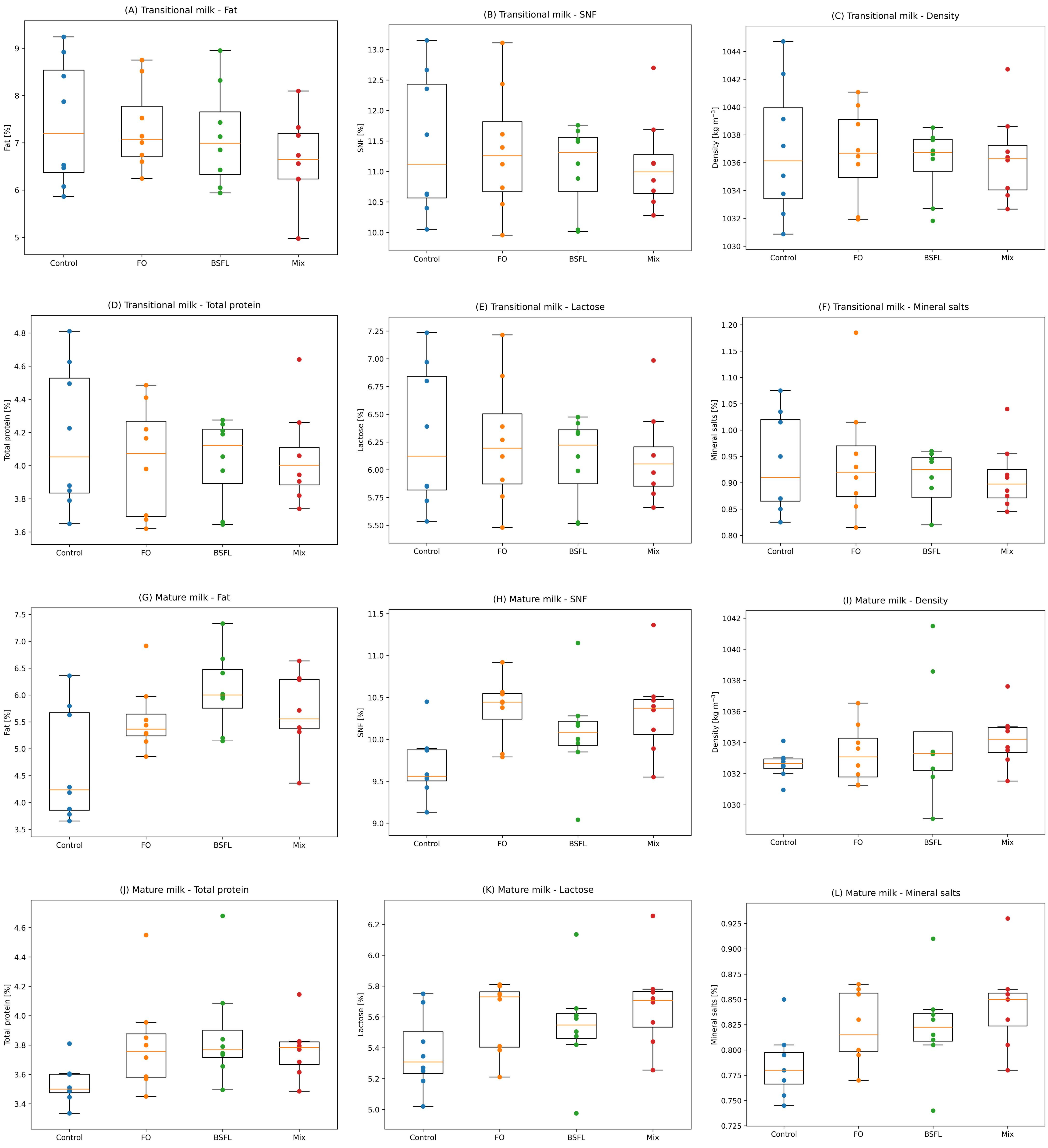

Figure S1. Ewe-level physicochemical composition of transitional and mature sheep milk. Panels A-F show transitional milk: fat (A), solids-not-fat (SNF; B), density (C), total protein (D), lactose (E), and mineral salts (F). Panels G-L show the corresponding variables in mature milk: fat (G), SNF (H), density (I), total protein (J), lactose (K), and mineral salts (L). Each point represents one ewe and is the mean of samples collected at 24 h and on day 3 postpartum for transitional milk, or on days 7 and 21 postpartum for mature milk (n = 8 ewes per treatment group). Boxes show the median and interquartile range; whiskers extend to the most extreme observations within 1.5 x IQR. All observations were retained. FO, flaxseed oil; BSFL, black soldier fly larvae oil; Mix, a 2:1 mixture of FO and BSFL oil.
